# Field and laboratory studies reveal interacting effects of stream oxygenation and warming on aquatic ectotherms

**DOI:** 10.1111/gcb.13240

**Published:** 2016-02-29

**Authors:** Wilco C.E.P. Verberk, Isabelle Durance, Ian P. Vaughan, Steve J. Ormerod

**Affiliations:** ^1^ Department of Animal Ecology and PhysiologyRadboud University NijmegenHeyendaalseweg, 1356525 AJNijmegenThe Netherlands; ^2^ Catchment Research GroupCardiff School of BiosciencesCardiff UniversityCardiffCF10 3AXUK

**Keywords:** climate change, hypoxia, insects, multiple stressors, pollution, streams, temperature, thermal tolerance

## Abstract

Aquatic ecological responses to climatic warming are complicated by interactions between thermal effects and other environmental stressors such as organic pollution and hypoxia. Laboratory experiments have demonstrated how oxygen limitation can set heat tolerance for some aquatic ectotherms, but only at unrealistic lethal temperatures and without field data to assess whether oxygen shortages might also underlie sublethal warming effects. Here, we test whether oxygen availability affects both lethal and nonlethal impacts of warming on two widespread Eurasian mayflies, *Ephemera danica*, Müller 1764 and *Serratella ignita* (Poda 1761). Mayfly nymphs are often a dominant component of the invertebrate assemblage in streams, and play a vital role in aquatic and riparian food webs. In the laboratory, lethal impacts of warming were assessed under three oxygen conditions. In the field, effects of oxygen availability on nonlethal impacts of warming were assessed from mayfly occurrence in 42 293 UK stream samples where water temperature and biochemical oxygen demand were measured. Oxygen limitation affected both lethal and sublethal impacts of warming in each species. Hypoxia lowered lethal limits by 5.5 °C (±2.13) and 8.2 °C (±0.62) for *E. danica* and *S. ignita* respectively. Field data confirmed the importance of oxygen limitation in warmer waters; poor oxygenation drastically reduced site occupancy, and reductions were especially pronounced under warm water conditions. Consequently, poor oxygenation lowered optimal stream temperatures for both species. The broad concordance shown here between laboratory results and extensive field data suggests that oxygen limitation not only impairs survival at thermal extremes but also restricts species abundance in the field at temperatures well below upper lethal limits. Stream oxygenation could thus control the vulnerability of aquatic ectotherms to global warming. Improving water oxygenation and reducing pollution can provide key facets of climate change adaptation for running waters.

## Introduction

Human dependency on ecosystem services provided by rivers and streams makes them one among the world's most important resources (Postel & Carpenter, 1997; Vörösmarty *et al*., 2010; Durance *et al*., 2016). Simultaneously, however, they are among the most vulnerable of all ecosystems to climate warming (see Durance & Ormerod, 2007; Vörösmarty *et al*., 2010; Carpenter *et al*., 2011; Isaak & Rieman, 2013). While many current studies attempt to address the consequences of such vulnerabilities by appraising the effects of changes in discharge or thermal regimes alone (Domisch *et al*., 2011; Isaak & Rieman, 2013), in reality climate risks to freshwaters arise through complex interactions with a wide range of other stressors (Ormerod *et al*., 2010). They include natural interactions among species that can render organisms more sensitive to warming effects (Durance & Ormerod, 2010); dispersal limitations stemming from the dendritic nature of rivers that reduces the capability of species to track climatically dependent shifts in habitat suitability (Clews *et al*., 2010; Isaak & Rieman, 2013; Jaeger *et al*., 2014); or stressors such as abstraction, eutrophication and organic pollution that might intensify climate‐change effects unless they are managed effectively (Durance & Ormerod, 2009; Galbraith *et al*., 2010; Verberk & Bilton, 2013; Piggott *et al*., 2015; Jenny *et al*., 2016). Improved mechanistic understanding of climate‐change interaction with these other stressors is urgently required for effective mitigation or adaptation to minimize warming impacts.

The interactions between warming, dissolved oxygen concentrations and organic pollution are particularly relevant as experimental work on aquatic ectotherms (Frederich & Pörtner, 2000; Pörtner *et al*., 2006; Eliason *et al*., 2011), including those in freshwaters (Moran *et al*., 2010; Verberk & Bilton, 2011, 2013), has shown how improved water quality and increased water oxygenation could actually offset adverse warming effects. Hypoxia and warming are believed to interact directly such that oxygen limitation sets the upper, lethal thermal limits of aquatic ectotherm species (Winterstein, 1905). High temperatures cause oxygen demand to increase beyond the ability of organisms to supply oxygen to their tissues, causing a mismatch between oxygen supply and demand (Pörtner & Knust, 2007; Pörtner, 2010; Verberk *et al*., 2011). At this point, anaerobic energy generation becomes important (Pörtner, 2010; Verberk *et al*., 2013; Sørensen *et al*., 2014). As a result, better oxygenation of the medium should improve heat tolerance, whereas lower temperatures improve hypoxia tolerance (Whitney, 1939; Knight & Gaufin, 1964; Pörtner *et al*., 2006; Woods *et al*., 2009; Verberk *et al*., 2013). So far, however, these interactions are understood mostly through experiments where temperatures exceed those experienced by species in the field and mortality was used as an endpoint. Consequently, it remains unknown whether good oxygenation can offset adverse warming effects under ecologically relevant temperatures and oxygen conditions, for example by enhancing survival or through effects on sublethal processes such as growth and reproduction.

Here, we address this key knowledge gap by investigating the interactive effects of oxygen limitation and temperature on organismal performance both experimentally in the laboratory and by analysing extensive field data. Stream invertebrates are an important component of river ecosystems, being a crucial link between primary producers, detritus pools or primary consumers, and predators higher up in the trophic hierarchy (Wootton & Power, 1993; Malmqvist, 2002). They play a key role in sediment transport dynamics (Statzner, 2012) and they are the main focus of biomonitoring programmes owing to their sensitivity to a range of anthropogenic stressors (Rosenberg & Resh, 1993). Specifically, we investigated the effect of oxygen availability on both lethal and nonlethal impacts of warming in two common species of mayfly, *Ephemera danica* Müller 1764 and *Serratella ignita* (Poda 1761). Mayfly nymphs are often a dominant component of the invertebrate assemblage in streams, and play a vital role in stream food webs, recycling organic detritus or grazing on periphyton and algae. In turn, both the nymphs and adults form an important food source for predatory invertebrates and fish, being often used as bait by anglers. In addition to their importance for aquatic consumers, emergence of adult mayflies from streams also constitutes a substantial export of benthic production to riparian consumers such as birds, bats, lizards and spiders (Sweeney & Vannote, 1982; Nakano & Murakami, 2001). In general, aquatic life stages are more vulnerable to the synergistic effects of warming and hypoxia (Verberk & Bilton, 2013; Verberk *et al*., 2016). Indeed, the two mayfly species investigated require well oxygenated waters for their larval development, making them ideal candidates to test for an interactive effect between temperature and oxygen. Testing these ideas about climate change effects on field populations has, in the past, been restricted by a scarcity of basic ecological data. However, extensive data on benthic macroinvertebrates and water chemistry in UK rivers now offer a major opportunity to assess responses of stream organisms to warming in interaction with other stressors (Environment Agency, 2002; Vaughan & Ormerod, 2012a). These data suggest strong improvement in water quality over two decades (Vaughan & Ormerod, 2012b), and present an important opportunity to examine interaction with long‐term climate warming over this time period (Durance & Ormerod, 2009).

We hypothesized that oxygen limitation would modulate thermal performance for acute lethal limits established during experiments as has been shown for other mayfly nymphs (Verberk & Bilton, 2013). Since sub‐lethal limits will necessarily be lower than lethal temperatures with concomitant lower oxygen requirements, oxygen limitation could be less problematic for sublethal limits. Nevertheless, while energy deficits in the short term may be remediated by recruiting anaerobic metabolism, organisms depend on oxygen and aerobic metabolism to meet energy demands associated with activities in the long term such as feeding, growth and reproduction. Consequently, chronic exposure to warm water could still invoke oxygen limitation to sustain these essential activities with knock‐on consequences for abundance and population persistence. We therefore test whether the occurrence of mayflies can be predicted better when including the interactive effects of oxygenation and stream temperature and if so whether the interaction is such that poor oxygenation exacerbates the negative impacts of stream warming.

## Materials and methods

### Study species

Like many other species of Ephemeroptera, Plecoptera and Trichoptera, the three dominant invertebrate orders in stream assemblages, the two mayflies studied are sensitive to organic effluents and eutrophication (Hawkes, 1997), reflecting their requirement for well‐oxygenated waters and potential susceptibility to oxygen shortage at high temperature. We also chose these species for pragmatic reasons because although the field data set was mostly resolved at family level, Ephemeridae are represented by only three species in UK and Ephemerellidae by only two species. For each mayfly family, the focal species is overwhelmingly the most common in their respective family, representing over 97% of the individuals in the 2500+ samples for which we had species‐level resolution. *Ephemera danica*, Müller 1764 is a large mayfly occupying the depositional zones of streams and rivers with a sandy bottom where the nymphs construct a tubular burrow in the sediment and feed by filtering or collecting fine particulate organic detritus. The species is usually semivoltine in UK, although in warmer waters populations may be univoltine (Bennett, 2007). The main flight period is towards the end of May, however adults are often present between April and November. *Serratella ignita* (Poda 1761) is typical of fast‐flowing rivers and streams where nymphs feed by collecting or gathering fine particulate organic detritus and algae from aquatic vegetation or stony substrata. The species is mainly univoltine in colder streams where it overwinters as diapausing eggs. In warmer streams, eggs may hatch before winter, giving rise to a separate winter and summer generation (Elliot *et al*., 1988), and the species there could be partially bivoltine (Rosillon, 1988). Adults occur from April to September.

### Thermal tolerance laboratory experiments

Mayfly nymphs for laboratory experiments were collected in spring (early May) from Torrington River, Devon, UK, ranging in fresh weight between 15 and 128 mg (*E. danica*) and between 2.0 and 11.6 mg (*S. ignita*). Nymphs were maintained in the laboratory at 10 ± 1 °C in a 12 L:12 D regime in aquaria containing artificial pond water, buffered and diluted to reflect the pH and conductivity of the field site. Before recording critical temperatures, all species were acclimated for at least 7 days to laboratory conditions.

To assess critical thermal maxima (*CT*_max_), we employed previously described methods (Verberk & Bilton, 2011; Verberk & Calosi, 2012). Individual nymphs (*n* = 18 for *E. danica* and *n* = 27 for *S. ignita*) were placed in flow‐through chambers and water was supplied to these chambers from a header tank after having passed through a tubular counter‐current heat exchanger. Water in the header tank was of the same composition as that used to maintain animals and was bubbled with a mixture of 20% oxygen and 80% nitrogen, obtained using a gas‐mixing pump (Wösthoff, Bochum, Germany). Individuals were left resting for 1 h at the equilibration temperature of 10 °C, after which temperature in the experimental chambers was increased by 0.25 °C min^−1^, using a Grant R5 water bath with a GP200 pump unit (Grant Instrument Ltd, Cambridge, UK), connected to the heat exchanger. Temperatures were logged using a HH806AU digital thermometer (Omega Engineering Inc., Stamford, CT, USA). Different sized flow‐through chambers were used for each species. *E. danica* was placed in larger chambers (70 × 70 × 30 mm) and provided with sand as burrowing substrate, which they readily used. *S. ignita* was placed in smaller cylindrical chambers (6 mm in diameter, 20 mm long) and their behaviour was observed under a magnifying glass. The amount of water passing through these flow‐through chambers was matched to their size. For the larger chambers containing *E. danica*, water was supplied to five chambers (total volume of 0.735 l) at a flow rates of 0.031–0.033 l per second, resulting in a refresh rate of 22–24 s. For the smaller chambers with *S. ignita*, water was supplied to each chamber individually at 0.21–0.22 ml per second, resulting in a refresh rate of 10–11 s.

*CT*_max_ is defined as the point at which an animal loses its ability to escape from conditions that will lead to its death (Lutterschmidt & Hutchison, 1997). During progressive warming, nymphs of *E. danica* first emerged from their burrowed position and began swimming (at about 6 °C below *CT*_max_). Loss of equilibrium occurred next as nymphs fell upon their backs, which was followed by the onset of spasms. After that, gill movement was no longer coordinated and faltered and this endpoint could be most reliably determined and is here taken as *CT*_max_. Similarly*, S. ignita* stopped ventilation and movement at *CT*_max_. Below *CT*_max_, larvae were inactive, until near the end of the trials, when they began to crawl, lose equilibrium and gill beating became intermittent shortly before stopping altogether at *CT*_max_.

*CT*_max_ was assessed at hypoxic (5 kPa), normoxic (20 kPa) and hyperoxic (60 kPa) conditions. Different levels of oxygenation were achieved by changing the oxygen–nitrogen gas mixture obtained using the gas‐mixing pump (Wösthoff). The gas mixture was adjusted 10 min after placing the animals in the small flow‐through chambers, to allow for gradual exposure to hypoxic and hyperoxic conditions during the 1 h resting period. To prevent equilibration with the atmosphere, the header tank was sealed using an 18 mm thick expanded polystyrene sheeting and other openings were closed off with plastic material. During the 1 h resting period, oxygen levels in the outflow water from the chambers were measured approximately every 15 min, to verify that the oxygen levels had stabilized to hypoxic, normoxic and hyperoxic conditions at the onset of warming. Because some equilibration with the atmosphere could not be prevented, nominal output values from the gas mixer were slightly more extreme (3 kPa for hypoxia and 65 kPa for hyperoxia) in order to achieve the desired oxygen conditions in the test chambers.

For each species, we used a GLM to test for an effect of experimental oxygen conditions (independent factor) on the observed thermal tolerance (dependent factor). The data had homogeneity of variances, but residuals were normally distributed only after excluding one outlier. Therefore, the data were also analysed by means of a non‐parametric Kruskal–Wallis test, with three pair‐wise comparisons using Mann–Whitney tests with a Bonferroni corrected alpha of 0.0167 (0.05/3). Full data (with outliers) are presented, along with the statistics for the nonparametric approach, but both approaches flagged the same contrasts as statistically significant.

### Field data

Field data collection followed a standardized protocol of 3‐minute kick‐sampling followed by predominantly family level identification in the laboratory (see Vaughan & Ormerod, 2012b; data from the Environment Agency and Natural Resources Wales). From the national database, locations were selected with monthly water chemistry and spot temperature sampling within 50 m of the kick sample location. Temperature and chemistry records up to 90 days prior to the kick sample and 30 days afterwards were averaged to represent the water chemistry and temperature associated with that sample. The presence or absence of *E. danica* and *S. ignita* was recorded within each sample, assuming all Ephemeridae and Ephermerellidae records belonged to these two species respectively, as discussed above. This resulted in a sample size of 42 293 kick samples with accompanying temperature and water chemistry from 2632 locations across England and Wales, covering the years 1989–2008 (Table 1). The pH was recorded to control for effects of acid‐base status on mayfly occurrences and Biochemical Oxygen Demand (BOD) was recorded using standard and quality controlled methods, to represent stream oxygenation. BOD values were represented more widely through our data than dissolved oxygen concentrations, but additional data analysis also revealed several potential benefits in using BOD (see Data S1). First, there was a clear negative relationship between BOD and dissolved oxygen concentrations (GLM: Beta = ‐0.97, t_1,2337_ = ‐17.85; *P* < 0.0001; Fig. S1). Second, temperature directly affects oxygen solubility in water, and hence oxygen concentrations (see Verberk *et al*., 2011), but affects BOD less; thus, BOD could be used more effectively in analyses that required us to account for thermal effects that were independent of oxygen concentrations (see Data S1). Third, BOD is likely to reflect potential deoxygenation in benthic microhabitats occupied by mayflies, where decomposing organic matters is often deposited, and dissolved oxygen concentrations are less readily measured by routine data collection or when oxygen minima occur (Macan, 1963). Finally, our additional analyses suggested that oxygen minima were particularly sensitive to increasing BOD values, this reflecting potentially more important limits on organisms than average values (see Data S1).

**Table 1:** Summary of the data set with average values for water quality characteristics and occupancy and abundance of each mayfly species

Variable	Value
pH (mean ± SD)	7.855 ± 0.376
Temperature (mean ± SD)	11.11 ± 3.52
BOD (mean ± SD)	2.01 ± 1.83
Relative temperature (mean ± SD)	−0.0029 ± 1.90
Number of samples	42 293
Number of sites	2632
Number of samples per site	16.07 ± 8.72
Number of sites occupied by *Ephemera danica* (%)	1168 (44.4)
Number of samples occupied by *E. danica* (%)	8283 (19.6)
Abundance class of *E. danica* in occupied sites (mean ± SD)	1.296 ± 0.494
Number of sites occupied by *Serratella ignita* (%)	1649 (62.7)
Number of samples occupied by *S. ignita* (%)	9436 (22.3)
Abundance class of *S. ignita* in occupied sites (mean ± SD)	1.544 ± 0.678

As the occurrence of both mayfly species differed regionally, we included region as a factor in our analysis, following the eight regions distinguished by the Environment Agency and Natural Resources Wales (Fig. S2): South West, South East, Thames, Midlands, Wales, Anglian, North East, North West. Although water temperatures in UK rivers can vary diurnally by 4 °C in spring and 2 °C in autumn, there was no systematic variation across sites in the time of day at which measurements were made (Webb & Zhang, 1999; Malcolm *et al*., 2008). Moreover, continuous water temperatures available from 9% of the sites showed that spot measurements were within <2° of true diurnal values in spring and within <1° in autumn. Finally, across the large number of samples these errors should average out.

The field data were analysed in two ways. First, to test a key prediction of our study, namely that in warmer sites, mayflies would place more stringent demands on water oxygenation, we compared the BOD values between sites, differentiating between cold and warm sites and whether sites were occupied by mayflies or not. Since water temperature varies seasonally, we calculated the *relative* water temperature for each sample, i.e. the difference between the water temperature of a given sample and the average water temperature for all samples in the same region in the same calendar month. Next, stream sites were classified as being relatively warm (2+ °C above average), relatively cold (2+ °C below average) or neither of these (ambient temperature). For each site, we calculated the 90th percentile of the BOD values across all sampling occasions at that site (i.e. spanning the 1989–2008 period).We used 90th percentiles, as population persistence is likely set by extremely low levels of oxygenation, rather than the average and 90th percentiles reflected a robust measure of BOD values, being resistant to the effects of outliers. A given site was considered occupied if mayflies were found in more than 25% of the samples, and unoccupied if the species was never detected. The 25% threshold was chosen to exclude locations where the focal species only occurred rarely/intermittently and also resulted in equal‐sized groups for occupied and unoccupied samples. A lower threshold for occurrence of 15% gave qualitatively identical results (data not shown). Differences in BOD values between the six ‘treatment’ groups (three temperature categories and the mayfly occurrence or absence) were tested using Tukey *posthoc* tests following a global test using GLM. A similar analysis was performed by classifying sites according to their level of oxygenation, with site oxygenation being excellent (BOD values of 1 and lower), good (BOD values between 1 and 2), or poor (BOD values >2) and testing for differences in stream temperature (again expressed as the 90th percentile).

In a second analysis, we tested for the presence of interactive effects between oxygen and temperature on mayfly occurrence, while also accounting for other sources of variation. We included region in our analysis to account for differences in regional distribution of both mayfly species. We similarly included pH as a factor as this is known to affect mayfly abundance (Durance & Ormerod, 2007). In this analysis, relative temperature, calculated as explained above, was analysed as a continuous factor. To test the key prediction that mayfly occurrence is best explained by models including the interaction between (relative) temperature and stream oxygenation (average BOD values), we performed a model comparison. We also considered the possibility that the importance of stream oxygenation differed across the eight regions. This generated four possible models, the simplest model lacking any interactions, the most complex model incorporating both interactions and two models each incorporating one or the other type of interaction (Table 2). To test for the main effects of oxygen and temperature, we also included a fifth model lacking these two factors. The Akaike's information criterion (AIC) of each model was calculated to assess the relative evidence in support of the alternative models. For the main effects of BOD, we allowed for nonlinearity in responses and included BOD as a second degree polynomial. To account for nonindependence of multiple samples at a given site, we averaged abiotic conditions per site and ran binomial GLMs with a binomial error distribution and a log link function to explain mayfly occurrence (number of samples where the mayfly occurred out of the total number of samples at a given site).

**Table 2:** Model comparison for the two studied species. Best model is highlighted in boldface

Species	Model specification	df	AIC	∆ AIC	Temperature × BOD (*z*‐value; *P*‐value)
*Ephemera danica* (proportion of occupied samples at a given site as dependent factor)	*E. danica* ~ Region + pH	2616	24 011.72	3221.71	–
	*E. danica* ~ BOD (2nd degree polynomial) + Temperature + Region + pH	2613	21 330.55	540.54	–
	*E. danica* ~ BOD (2nd degree polynomial) + Temperature + Region + pH + Region × BOD	2606	20 887.48	97.47	–
	*E. danica* ~ BOD (2nd degree polynomial) + Temperature + Region + pH + Temperature × BOD	2612	21 265.80	475.79	−8.309; <0.0001
	***E. danica*** ** ˜ BOD (2nd degree polynomial) + Temperature + Region + pH + Temperature × BOD + Region × BOD**	**2605**	**20 790.01**	**0.00**	**−10.249; <0.0001**
*Serratella ignita* (proportion of occupied samples at a given site as dependent factor)	*S. ignita* ~ Region + pH	2616	17 279.74	2843.87	–
	*S. ignita* ~ BOD (2nd degree polynomial) + Temperature + Region + pH	2613	14 974.96	539.09	–
	*S. ignita* ~ BOD (2nd degree polynomial) + Temperature + Region + pH + Region × BOD	2606	14 513.26	77.39	–
	*S. ignita* ~ BOD (2nd degree polynomial) + Temperature + Region + pH + Temperature × BOD	2612	14 926.12	490.25	−7.214; <0.0001
	*S. **ignita*** ** ˜ BOD (2nd degree polynomial) + Temperature + Region + pH + Temperature × BOD + Region × BOD**	**2605**	**14 435.87**	**0.00**	**−8.909; <0.0001**

As in the first analysis, abiotic conditions were averaged across all sampling occasions at that site (i.e. spanning the 1989–2008 period). Splitting the data in two equal time periods (pre and post 1997) and rerunning the same analyses for each time period did not qualitatively alter our results, so we chose to present those on the whole time period. The binomial analysis on mayfly occurrence negated the possibility to model mayfly abundances, but we tested whether average abundance at given site was strongly correlated with the proportion of occupied samples at a given site, as is commonly reflected in the relationship between abundance and occupancy (e.g. Verberk *et al*., 2010). This proved to be the case for each of the two species (*E. danica*: Adj. *R* ^2^ = 0.88; *P* < 0.0001; *S. ignita*: Adj. *R* ^2^ = 0.76; *P* < 0.0001). Also, we reran the same analyses on the data excluding data from the summer months (June, July, August), as the emergence of nymphs could have confounded the data on abundances. This approach confirmed the results of the main binomial GLM reported in the main text as the occurrence of both mayflies remained to be negatively impacted by the interactive effects of temperature and oxygen (Table S3 and S4). All analyses were performed using R (R‐Development‐Core‐Team 2013).

## Results

**Figure 1:**
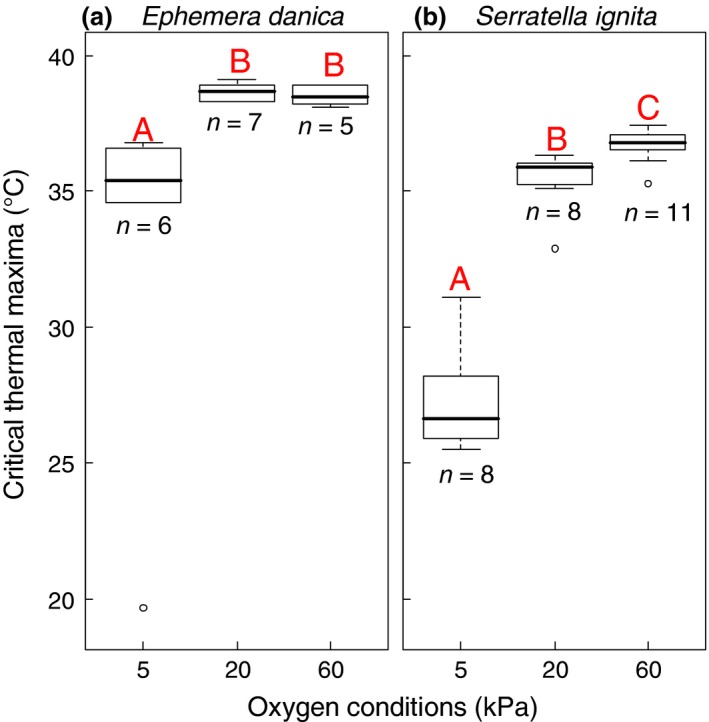
Critical temperatures measured by in *Ephemera danica* (a) and in *Serratella ignita* (b) at the three oxygen conditions. Letters indicate significant differences among oxygen conditions, which are based on pair‐wise comparisons using Mann–Whitney tests, employing an alpha of 0.01667 (Bonferroni corrected for three pair‐wise comparisons).

Experimental manipulation demonstrated that water oxygenation significantly affected the heat tolerance in mayfly nymphs of *E. danica* (Fig. 1a) and especially *S*. *ignita* (Fig. 1b) (Kruskal–Wallis test: *χ* ^2^ = 11.78; *P *=* *0.0028 for *E. danica* and *χ* ^2^ = 20.77; *P *<* *0.0001 for *S. ignita*). For *E. danica*, mean values for *CT*_max_ (±SD) were 5.5 °C (±2.13) higher in normoxia relative to hypoxia (median values differed by 3.3 °C). The one outlier can be readily explained as this concerned a nymph which was close to ecdysis, a critical stage during which old tracheal linings are shed, and the resulting impairment of respiration (Camp *et al*., 2014) explains the reduced heat tolerance of this individual at hypoxia (19.7 °C). In *S. ignita* heat tolerance was improved by a staggering 8.2 °C (±0.62) in normoxia relative to hypoxia (median values differed by 9.3 °C), while hyperoxia even further improved heat tolerance by 1.2 °C (±0.57) (median values differed by 0.9 °C).

**Figure 2:**
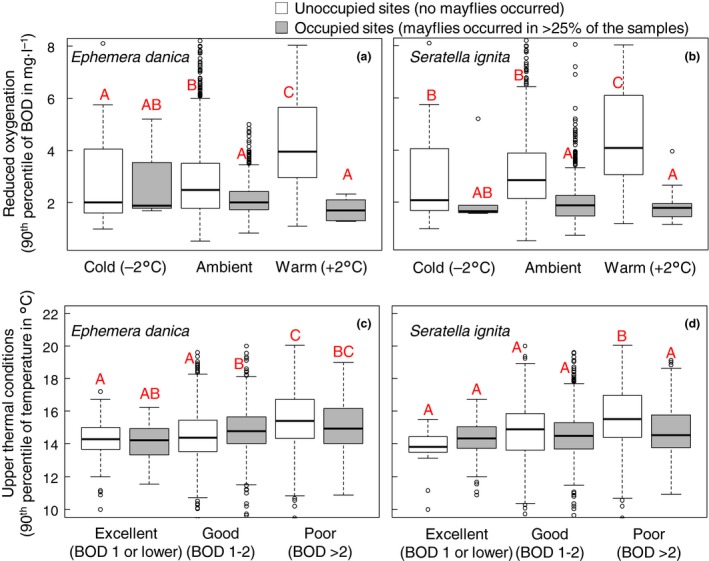
Differences in oxygenation and temperature conditions between unoccupied sites and sites that are occupied by *Ephemera danica* (a, c) and *Serratella ignita* (b, d). Boxplot show the biological oxygen demand (BOD) values (a, b) and stream temperature (c, d) of the 42 293 samples calculated as the 90th percentile for each site. Sites were grouped into three different categories based on BOD threshold values and stream temperature and were classified as occupied when mayflies were recorded at more than 25% of the samples at a given site. Sites where mayflies were never recorded are classified as unoccupied sites.

**Figure 3:**
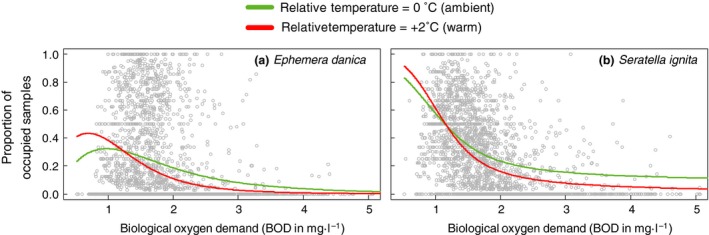
Projected model responses of *Ephemera danica* (a) and *Serratella ignita* (b).occupancy (proportion of samples where species was present at a given site) along a gradient of deteriorating oxygenation (increasing BOD‐values). Model responses are calculated for ambient temperatures and warm temperatures (relative temperature +2). Lines represent the average across all eight regions. Model summaries are given in Table 2 and Table S1 and S2.

Field data provided further evidence that both mayfly species require greater water oxygenation under warmer conditions. Oxygenation was generally better at stream sites where the mayflies were present compared to stream sites where they were absent (Fig. 2) illustrating the requirement for well oxygenated water for both species. For both *E. danica* (Fig. 2a) and *S. ignita* (Fig. 2b), this difference in BOD value between occupied and unoccupied sites was greatest in warm sites (sites where stream temperatures were at least 2 °C above the mean). Though less pronounced due to greater within‐site variability in temperature, a similar pattern was found for differences in water temperature between occupied and unoccupied sites. Cooler sites were occupied when oxygenation was poor; *E. danica* could occupy warmer sites provided that these were well oxygenated (90th Percentile BOD values between 1 and 2) but did no longer do so when oxygenation was poor (90th Percentile OD values >2) (Fig. 2c). Similarly, *S. ignita* occupied cooler sites when oxygenation was poor (Fig. 2d). These interactive effects of temperature and stream oxygenation proved important for both mayflies as the interaction term was always retained in the best models for site occupancy (Table 2; and Table S3 and S4). Site occupancy, expressed as the proportion of occupied samples at a given site, generally declined rapidly with increasing BOD, and this decline was steeper under warmer conditions (Fig. 3) for both species. There was some regional variation, with site occupancy being much less responsive to deteriorating oxygenation in the South West, Wales and the North West, but also in these regions did site occupancy decline more steeply under warmer conditions (see Fig. S3).

## Discussion

A mechanistic understanding is essential to predict the vulnerability of aquatic organisms to global warming and to guide management efforts aimed at climate change adaptation (Thomas *et al*., 2016). Our experimental manipulation indicated that oxygen limitation can potentially constrain experimental heat tolerance limits for two widespread and abundant mayflies, *Ephemera danica* and *Serratella ignita* (Fig. 3). Effects of oxygen on heat tolerance were nonlinear, being especially important under hypoxia (see also Verberk & Calosi, 2012), although hyperoxia did improve heat tolerance in *S. ignita*. Importantly, we also found interactive effects of oxygen and temperature on their occurrences under field conditions (Fig. 2). Our study thus bridges a gap, showing that the mechanistic understanding gained in our short term laboratory experimentation has relevant consequences at ecological time scales, which in the case of the semivoltine species *E. danica* extends across multiple years.

In our analysis, BOD values were taken to indicate the risk of species experiencing hypoxic conditions: our models consistently showed strong reductions in abundance for both species as BOD doubled from 1 to 2 mg l^−1^, which were exacerbated when stream temperatures were high. These differences are ecologically very relevant given that the mean BOD value in UK streams during this study was around 2 mg l^−1^. Many streams had BOD values in excess of 2 mg l^−1^ and improvements in stream oxygenation over this range have been previously shown to allow the recovery or persistence of cool‐water species in English chalk streams despite warming (Durance & Ormerod, 2009). We chose BOD as a surrogate measure for stream oxygen conditions partly because data availability was better, and validated this selection analytically (see Methods and Data S1). Importantly, BOD values are also likely to reflect potential oxygen deficit in benthic microhabitats used by mayflies caused by the deposition of decomposing organic matter (see Data S1). Moreover, dissolved oxygen values are not only more variable and diurnally linked to photosynthesis (Macan, 1963), but saturation oxygen concentrations are also linked directly to temperature (Dejours, 1981; Verberk *et al*., 2011). The use of BOD to indicate oxygenation is not without constraints and for example under turbulent flow and with considerable reaeration, even high BOD values may be uncoupled from oxygen stress. Indeed, our model results showed that the combined effect of warming and deteriorating water oxygenation (higher BOD values) on mayfly occurrences was less of a problem in upland regions with fast‐flowing streams (Wales and Southwest England). However, these effects are important in providing additional confirmation of the overall principle we demonstrate in this paper: in streams where oxygen stress is mitigated, combined effects of warming and oxygen stress are less apparent than elsewhere. These effects also suggest that instead of using BOD as a proxy for possible oxygen stress, better measurements of the oxygen conditions actually experienced by benthic organisms would further strengthen the patterns reported here.

In addition to demonstrating that an interaction between temperature and oxygen applies to both species investigated, there were also inter‐specific differences. In *E. danica*, critical lethal temperatures were higher than those found for *S. ignita* under all oxygen conditions. Compared to *S. ignita*, oxygen conditions also had smaller effects on the lethal temperatures of *E. danica*, even though these were still reduced by more than 5 °C under hypoxia (Fig. 2). Similarly, hyperoxia improved thermal tolerance in *S. ignita*, but not *E. danica*. Verberk & Bilton (2013) demonstrated that mayflies with poor ventilatory ability were more vulnerable to the combined effects of heat and hypoxia. Thus, *S. ignita* is apparently less able to meet increased oxygen demand under the experimental heating conditions, and oxygen delivery to its tissues seems already compromised under normoxic conditions in warm water. Possibly, *S. ignita* relies more on behavioural changes to upregulate oxygen uptake: This species lives on the substrate, rather than being buried in the stream bed, giving it the option to upregulate its oxygen uptake by selecting exposed microhabitats where rapid water flows reduce boundary layer thickness and enhance gas exchange (e.g. Statzner & Holm, 1982; Verberk & Atkinson, 2013).

The concordant effect of hypoxia on the manifestation of sublethal and lethal impacts of warming indicates that oxygen limitation not only sets limits to survival during acute heat stress as tested in our experiments, but may also mediate sublethal effects, restricting occurrence under warm conditions in field situations. It was recently suggested that there is a trade‐off between the ability of organisms to tolerate acute thermal stress and chronic thermal stress (Rezende *et al*., 2014). Indeed, in our case, *E. danica* was more tolerant of acute heat exposure than *S. ignita*, but optimal temperatures of field abundances were lower, as the average stream temperature weighed by abundance was 10.8 °C for *E. danica*, vs. 11.7 °C for *S. ignita*. Given such a trade‐off, the extent to which oxygen limits thermal tolerance may differ between acute heat stress during experimental heating trials and chronic thermal stress under field conditions. While the BOD values in the field cannot be related directly to the oxygen tensions used in the experiment, a case could be made that on longer timescales good oxygenation becomes more important for offsetting detrimental effects of warming. Using the Environmental Agency's General Quality Assessment of rivers, the experimental hypoxia treatment which reduced thermal limits by 3 and 9 °C for the two mayfly species, would be equivalent to a BOD value between 8 and 15 mg l^−1^. In contrast, mayfly occurrences in the field are largely already affected within the range of BOD values 1–2 mg l^−1^, and it is within this range that a 2 °C warming has strong exacerbating effects. Thus, oxygen stress is important for acute and – possibly even more so – for chronic thermal stress.

Oxygen is crucial for aquatic organisms to maintain ATP levels and survive warming as aerobic metabolism generates about 15‐fold more energy compared to anaerobic metabolism. While previous studies found that warming effects were offset by improvements in water quality (Durance & Ormerod, 2009; Vaughan & Ormerod, 2014), here we link these two directly and show that they interact, with changes in BOD of 1 unit being problematic especially when combined with warming effects of 2°. Oxygen limitation is more likely to occur in aquatic ectotherms which rely on underwater gas exchange than in aerial gas exchangers (Verberk & Atkinson, 2013; Verberk *et al*., 2016), as the extent to which oxygen limits thermal tolerance in aquatic ectotherms has been shown to depend on their ability to regulate gas exchange (Verberk & Bilton, 2013, 2015; Koopman *et al*., 2016). Species that are poor at regulating oxygen uptake (e.g. gill breathers and tegument breathers) showed consistently reduced heat tolerance under hypoxia, whereas species that breathe air, having good regulatory ability, were much less affected by hypoxia. As the adult stage is terrestrial and breathes atmospheric oxygen, oxygen limitation at warm temperatures is far less likely in adults (Giomi *et al*., 2014; Verberk & Bilton, 2015). However, at least at the nymphal stage, oxygen appears to limit the thermal tolerance of these mayfly species, echoing the message of Bartolini *et al*. (2013) that climate change effects are mediated by the most vulnerable life stages. As many species of aquatic macroinvertebrates rely on gills and tegument for their oxygen uptake, oxygen shortage may be a master variable controlling the vulnerability to global warming under field circumstances in a wide variety of aquatic ectotherms.

Our results provide highly relevant information for addressing climate change effects on freshwater communities and the ecosystem services they provide. Alongside methods to depress temperature gain and increase resilience (Broadmeadow *et al*., 2011; Thomas *et al*., 2016), improved water quality provides a key facet of climate change adaptation for running waters in reducing the multistressor threat of warming and organic pollution. Given the mechanistic underpinning of warming and hypoxia, such an adaptation strategy would also work for eutrophication, another major and ongoing stressor in both marine and freshwater systems (Diaz & Rosenberg, 1995; Dudgeon *et al*., 2006; Jenny *et al*., 2016), whose negative impact is largely through resulting hypoxia when increased algal biomass depletes oxygen concentrations during darkness. Reducing deoxygenation effects in surface waters could have further benefits when used in conjunction with stream shading as key adaptation strategies. It is increasingly unlikely that further global warming cannot be evaded, but evidence suggests that it may be possible to reduce its impacts when water oxygenation is maintained or improved. This could be achieved by reducing organic effluents and eutrophication, or by enhancing aeration through increasing base flow and via morphological alterations (pool‐riffle systems, tree roots). These may be important generic principles for other aquatic ectotherms and aquatic ecosystems.

## Supporting information


**Data S1.** Supporting analysis: investigating the link between dissolved oxygen and BOD.
**Figure S1.** Relationship between dissolved oxygen (DO) and biochemical oxygen demand (BOD).
**Figure S2.** Location of the 2632 sample sites divided over the eight EA regions distinguished.
**Figure S3.** Projected model responses of *Ephemera danica* (left) and *Serratella ignita* (right) occupancy (proportion of samples where species was present at a given site) along a gradient of deteriorating oxygenation (increasing BOD‐values).
**Table S1.** Model summary for *Ephemera danica* (best fitted model in Table 2).
**Table S2.** Model summary for *Serratella ignita* (best fitted model in Table 2).
**Table S3.** Model summary for *E. danica*, excluding summer months (June, July, August).
**Table S4.** Model summary for *S. ignita*, excluding summer months (June, July, August).
