# Supplementary material for: Field and laboratory studies reveal interacting effects of stream oxygenation and warming on aquatic ectotherms
Source: Glob Chang Biol. 2016 Feb 29;22(5):1769–78. doi: 10.1111/gcb.13240 (PMC5324560; doi:10.1111/gcb.13240)
Supplement: Supplementary file 1 — Data S1. Supporting analysis: investigating the link between dissolved oxygen and BOD. Figure S1. Relationship between dissolved oxygen (DO) and biochemical oxygen demand (BOD). Figure S2. Location of the 2632 sample sites divided over the eight EA regions distinguished. Figure S3. Projected model responses of Ephemera danica (left) and Serratella ignita (right) occupancy (proportion of samples where species was present at a given site) along a gradient of deteriorating oxygenation (increasing BOD‐values). Table S1. Model summary for Ephemera danica (best fitted model in Table 2). Table S2. Model summary for Serratella ignita (best fitted model in Table 2). Table S3. Model summary for E. danica, excluding summer months (June, July, August). Table S4. Model summary for S. ignita, excluding summer months (June, July, August). [file GCB-22-1769-s001.docx]

Supporting information captions

**Supporting Analysis**: investigating the link between dissolved oxygen and BOD

**Table S1**: Model summary for *Ephemera danica* (best fitted model in Table 2).

**Table S2**: Model summary for *Serratella ignita* (best fitted model in Table 2).

**Table S3**: Model summary for *E. danica*, excluding summer months (June, July, August).

**Table S4**: Model summary for *S. ignita*, excluding summer months (June, July, August).

**Figure S1**: Relationship between dissolved oxygen (DO) and biochemical oxygen demand (BOD). Shaded areas indicate confidence intervals. Plot is based on median values across the 2,341 sites for which coupled data on BOD and DO were available.

**Figure S2**: Location of the 2632 sample sites divided over the 8 EA regions distinguished.

**Figure S3**: Projected model responses of *Ephemera danica* (left) and *Serratella ignita* (right) occupancy (proportion of samples where species was present at a given site) along a gradient of deteriorating oxygenation (increasing BOD-values). Model responses are calculated for ambient temperatures and warm temperatures (relative temperature +2). Dashed lines represent the responses in the Southwest Region only. For reasons of comparison, the average across all eight regions (shown in Figure 3), are repeated here as thin lines.

Supporting information

**Supporting Analysis**: investigating the link between dissolved oxygen and BOD

We investigated the link between dissolved oxygen concentrations (DO) and Biochemical Oxygen Demand (BOD) values, while accounting for other physico-chemical variables (i.e. stream temperature and pH). Median values for all four determinants were calculated for each sample site. Dissolved oxygen was not recorded at all locations used in the main study, resulting in a dataset containing 2,341 sites with coupled data on (median) stream oxygenation (BOD and DO), water temperature and pH. Prior to analyses, BOD data were log-transformed. General linear models (GLMs) were used to model DO as a function of BOD and the other physico-chemical variables.

These analyses confirmed that there was a clear negative relationship between BOD and DO (GLM: Beta = -0.97, t_1,2337_ = -17.85; P<0.0001; Fig. S1). The strength of this relationship differed regionally, and was slightly weaker for upland regions with fast-flowing streams (Wales and Southwest England; Beta = -0.78, t_1,2336_ = -6.15; P<0.0001) than in the other regions (Beta = -0.98, t_1,236_ = -17.94; P<0.0001), but was always highly significant (Figure S1).

While both DO (Beta = -0.30, t_1, 2337_ = -27.32; P<0.0001) and BOD were affected by temperature (Beta = -0.031, t_1, 2337_ = -7.22; P<0.0001), the variance explained by temperature was much lower in the latter. We also performed a similar analysis that examined relationships between BOD and the 10^th^ percentiles for DO rather than median values to provide an approximation for DO minima. In this analysis, the relationship between BOD and DO 10^th^ percentiles was significantly steeper (Beta = -1.97, t_1,2337_ = -25,40; P<0.0001) than when using median values for DO (Beta = -0.97, t_1,2337_ = -17.85; P<0.0001). Thus, oxygen minima declined more rapidly with increasing BOD values than did the average values, and may therefore capture the extremes to which stream invertebrates are exposed.

Figure S1. Relationship between dissolved oxygen (DO) and biochemical oxygen demand (BOD). Shaded areas indicate confidence intervals. Plot is based on median values across the 2,341 sites for which coupled data on BOD and DO were available.

Table S1: Model summary of site occupancy for *E. danica* (best fitted model in Table 2), calculated as the ratio between number of samples occupied for a given site and the total number of samples for that site. The interaction between temperature (relative temperature) and oxygen (BOD) is highlighted in red.

Call:

glm(formula = Yd ~ poly(logBOD, 2) + relT + relT:logBOD + Reg:logBOD +

Reg + pH, family = binomial, data = D)

Deviance Residuals:

Min 1Q Median 3Q Max

-7.9997 -2.0397 -0.9054 0.6251 10.8164

Coefficients:

Estimate Std. Error z value Pr(>|z|)

(Intercept) -18.06614 0.50479 -35.789 < 2e-16 ***

poly(logBOD, 2)1 -109.11279 4.62671 -23.583 < 2e-16 ***

poly(logBOD, 2)2 -30.81057 3.07057 -10.034 < 2e-16 ***

relT 0.16134 0.04263 3.785 0.000154 ***

RegMI -0.49002 0.12639 -3.877 0.000106 ***

RegNE -0.93583 0.23753 -3.940 8.16e-05 ***

RegNW -2.59212 0.24271 -10.680 < 2e-16 ***

RegSO 1.03888 0.15285 6.797 1.07e-11 ***

RegSW -0.61503 0.13206 -4.657 3.20e-06 ***

RegTH 0.87812 0.16335 5.376 7.63e-08 ***

RegWA -0.99427 0.13047 -7.621 2.53e-14 ***

pH 1.94902 0.06238 31.245 < 2e-16 ***

relT:logBOD -1.70792 0.16664 -10.249 < 2e-16 ***

logBOD:RegMI 6.65857 0.56131 11.863 < 2e-16 ***

logBOD:RegNE 6.41969 1.00550 6.385 1.72e-10 ***

logBOD:RegNW 10.00630 0.94067 10.637 < 2e-16 ***

logBOD:RegSO 5.19479 0.66149 7.853 4.06e-15 ***

logBOD:RegSW 11.99657 0.61143 19.620 < 2e-16 ***

logBOD:RegTH 3.29547 0.73434 4.488 7.20e-06 ***

logBOD:RegWA 5.10321 0.82922 6.154 7.55e-10 ***

---

Signif. codes: 0 ‘***’ 0.001 ‘**’ 0.01 ‘*’ 0.05 ‘.’ 0.1 ‘ ’ 1

(Dispersion parameter for binomial family taken to be 1)

Table S2: Model summary of site occupancy for *S. ignita* (best fitted model in Table 2), calculated as the ratio between number of samples occupied for a given site and the total number of samples for that site. The interaction between temperature (relative temperature) and oxygen (BOD) is highlighted in red.

Call:

glm(formula = Yi ~ poly(logBOD, 2) + relT + relT:logBOD + Reg:logBOD +

Reg + pH, family = binomial, data = D)

Deviance Residuals:

Min 1Q Median 3Q Max

-5.3994 -1.7467 -0.5305 0.8967 9.4889

Coefficients:

Estimate Std. Error z value Pr(>|z|)

(Intercept) -10.77662 0.38610 -27.912 < 2e-16 ***

poly(logBOD, 2)1 -80.29068 4.74001 -16.939 < 2e-16 ***

poly(logBOD, 2)2 1.11179 1.34489 0.827 0.408420

relT 0.08334 0.03363 2.478 0.013215 *

RegMI 0.22965 0.13047 1.760 0.078389 .

RegNE 0.91533 0.19311 4.740 2.14e-06 ***

RegNW 0.57558 0.15211 3.784 0.000154 ***

RegSO 1.66468 0.16131 10.320 < 2e-16 ***

RegSW 1.05367 0.13121 8.030 9.71e-16 ***

RegTH 2.11314 0.18798 11.241 < 2e-16 ***

RegWA 0.77310 0.12513 6.178 6.47e-10 ***

pH 1.01363 0.04764 21.278 < 2e-16 ***

relT:logBOD -1.17733 0.13215 -8.909 < 2e-16 ***

logBOD:RegMI 4.55593 0.58068 7.846 4.30e-15 ***

logBOD:RegNE 3.15701 0.82084 3.846 0.000120 ***

logBOD:RegNW 4.63778 0.65633 7.066 1.59e-12 ***

logBOD:RegSO 1.50150 0.71601 2.097 0.035989 *

logBOD:RegSW 7.82489 0.61049 12.817 < 2e-16 ***

logBOD:RegTH -3.36483 0.89410 -3.763 0.000168 ***

logBOD:RegWA 8.46187 0.63620 13.301 < 2e-16 ***

---

Signif. codes: 0 ‘***’ 0.001 ‘**’ 0.01 ‘*’ 0.05 ‘.’ 0.1 ‘ ’ 1

(Dispersion parameter for binomial family taken to be 1)

Null deviance: 17829.9 on 2624 degrees of freedom

Residual deviance: 9984.8 on 2605 degrees of freedom

AIC: 14436

Number of Fisher Scoring iterations: 5

Table S3: Model summary of site occupancy for *E. danica*, excluding summer months (June, July, August), calculated as the ratio between number of samples occupied for a given site and the total number of samples for that site. The interaction between temperature (relative temperature) and oxygen (BOD) is highlighted in red.

Call:

glm(formula = Yd ~ poly(logBOD, 2) + relT + relT:logBOD + Reg:logBOD +

Reg + pH, family = binomial, data = D)

Deviance Residuals:

Min 1Q Median 3Q Max

-7.3834 -1.9211 -0.8579 0.6668 9.2418

Coefficients:

Estimate Std. Error z value Pr(>|z|)

(Intercept) -18.54017 0.53052 -34.947 < 2e-16 ***

poly(logBOD, 2)1 -120.74270 5.15614 -23.417 < 2e-16 ***

poly(logBOD, 2)2 -35.26775 3.35639 -10.508 < 2e-16 ***

relT 0.07685 0.04435 1.733 0.083101 .

RegMI -0.75129 0.13397 -5.608 2.05e-08 ***

RegNE -0.94946 0.24875 -3.817 0.000135 ***

RegNW -2.64104 0.25145 -10.503 < 2e-16 ***

RegSO 0.73466 0.15804 4.649 3.34e-06 ***

RegSW -0.84221 0.13749 -6.126 9.03e-10 ***

RegTH 0.46632 0.16003 2.914 0.003569 **

RegWA -1.00994 0.13420 -7.526 5.25e-14 ***

pH 1.97800 0.06549 30.203 < 2e-16 ***

relT:logBOD -1.44400 0.18368 -7.861 3.80e-15 ***

logBOD:RegMI 8.08723 0.62850 12.867 < 2e-16 ***

logBOD:RegNE 7.36814 1.07915 6.828 8.63e-12 ***

logBOD:RegNW 11.29416 1.01987 11.074 < 2e-16 ***

logBOD:RegSO 6.94394 0.71656 9.691 < 2e-16 ***

logBOD:RegSW 13.19019 0.66439 19.853 < 2e-16 ***

logBOD:RegTH 4.49297 0.78719 5.708 1.15e-08 ***

logBOD:RegWA 5.95220 0.87867 6.774 1.25e-11 ***

---

Signif. codes: 0 ‘***’ 0.001 ‘**’ 0.01 ‘*’ 0.05 ‘.’ 0.1 ‘ ’ 1

(Dispersion parameter for binomial family taken to be 1)

Null deviance: 20630 on 2624 degrees of freedom

Residual deviance: 14629 on 2605 degrees of freedom

AIC: 17693

Number of Fisher Scoring iterations: 6

Table S4: Model summary of site occupancy for *S. ignita*, excluding summer months (June, July, August), calculated as the ratio between number of samples occupied for a given site and the total number of samples for that site. The interaction between temperature (relative temperature) and oxygen (BOD) is highlighted in red.

Call:

glm(formula = Yi ~ poly(logBOD, 2) + relT + relT:logBOD + Reg:logBOD +

Reg + pH, family = binomial, data = D)

Deviance Residuals:

Min 1Q Median 3Q Max

-4.4931 -1.5017 -0.5475 0.7181 8.9219

Coefficients:

Estimate Std. Error z value Pr(>|z|)

(Intercept) -12.31786 0.42897 -28.715 < 2e-16 ***

poly(logBOD, 2)1 -92.64431 6.09372 -15.203 < 2e-16 ***

poly(logBOD, 2)2 1.26144 1.38191 0.913 0.36134

relT 0.06007 0.03533 1.701 0.08902 .

RegMI 0.04544 0.15618 0.291 0.77108

RegNE 0.69749 0.21710 3.213 0.00131 **

RegNW 0.53935 0.18015 2.994 0.00275 **

RegSO 1.64550 0.18239 9.022 < 2e-16 ***

RegSW 0.72593 0.15291 4.747 2.06e-06 ***

RegTH 1.55734 0.19591 7.949 1.88e-15 ***

RegWA 0.80846 0.14615 5.532 3.17e-08 ***

pH 1.13780 0.05271 21.588 < 2e-16 ***

relT:logBOD -0.97629 0.15082 -6.473 9.60e-11 ***

logBOD:RegMI 5.65835 0.75478 7.497 6.55e-14 ***

logBOD:RegNE 5.39015 0.96959 5.559 2.71e-08 ***

logBOD:RegNW 5.28195 0.85267 6.195 5.84e-10 ***

logBOD:RegSO 2.77265 0.87376 3.173 0.00151 **

logBOD:RegSW 9.86309 0.76600 12.876 < 2e-16 ***

logBOD:RegTH -1.39307 1.04254 -1.336 0.18148

logBOD:RegWA 10.05667 0.77879 12.913 < 2e-16 ***

---

Signif. codes: 0 ‘***’ 0.001 ‘**’ 0.01 ‘*’ 0.05 ‘.’ 0.1 ‘ ’ 1

(Dispersion parameter for binomial family taken to be 1)

Null deviance: 14535.4 on 2624 degrees of freedom

Residual deviance: 7747.7 on 2605 degrees of freedom

AIC: 11840

Number of Fisher Scoring iterations: 5

Figure S2. Location of the 2632 sample sites divided over the 8 EA regions distinguished.

Figure S3. Projected model responses of *Ephemera danica* (left) and *Serratella ignita* (right).occupancy (proportion of samples where species was present at a given site) along a gradient of deteriorating oxygenation (increasing BOD-values). Model responses are calculated for ambient temperatures and warm temperatures (relative temperature +2). Dashed lines represent the responses in the Southwest Region only. For reasons of comparison, the average across all eight regions (shown in Figure 3), are repeated here as thin lines.
